# Impact of (forensic) expert opinions according to the Istanbul Protocol in Germany—results and insights of the in:Fo-project

**DOI:** 10.1007/s00414-023-02950-1

**Published:** 2023-02-14

**Authors:** M. Jühling, L. M. König, H. Gruber, V. Wolf, St. Ritz-Timme, F. Mayer

**Affiliations:** 1 Institute of Legal Medicine, University Hospital Düsseldorf, Düsseldorf, Germany; 2 Department of Psychology, University of Cologne, Cologne, Germany; 3 Psychosocial Center for Refugees Düsseldorf e. V., Düsseldorf, Germany

**Keywords:** Torture, Istanbul Protocol, Interdisciplinarity, Clarification of facts, Asylum

## Abstract

As part of a third-party funded project, expert opinions according to the Istanbul Protocol (IP) standards were compiled in Germany on a larger scale for the first time. The assessment process was initiated for 130 project participants. Statistical analysis on numerous variables was performed to test the impact of the expert opinions, foremost of the forensic medical expert opinions, on the individuals’ asylum proceedings. The variables were drawn from forensic medical expert opinions and reports of findings, questionnaires for the study participants’ counsellors in the project and a query on the asylum status of the study participants. Regression analysis and bivariate analysis on two dependent variables—subjective impact on the asylum procedure from the counsellors’ point of view and objective change in the asylum status—were performed to test for an influence on asylum procedures. No statistically significant results were obtained for the objective change of the study participants’ asylum status. For the subjective dependent variable, a positive prediction was possible when simultaneously controlling for the independent variables *introduction of a forensic medical expert opinion* and *highest IP grade*; a negative prediction was possible when simultaneously controlling for the independent variables *introduction of a forensic medical expert opinion* and *use of IP grading*. Apart from the statistical analysis, a positive effect of the assessment on the psychosocial well-being of the study participants could be demonstrated. The results differed from other European studies which demonstrated a correlation between the objective outcome of an asylum procedure (asylum status) and, for example, specific types of violence or the number of documented injuries. Differences also occurred in the use of the plausibility grades proposed by the IP, which questions their use in cases in which the reported torture happened a relevant time ago. Therefore, compiling individually worded evaluations instead of using the IP grading system—if possible, by an experienced forensic physician—is recommended in this scenario. Still, the assessment of alleged torture experiences should follow the IP guidelines, since psychological assessments are of especially high importance in cases with healed physical injuries and since the results also demonstrated a positive effect on the psychosocial well-being of the study participants.

## Introduction

Torture remains a widespread practice employed by many (para-)governmental actors to subjugate, terrorize and/or dehumanize other persons [1].

The European Union has addressed this issue with the so-called Reception Directive [2] stating that asylum applicants, belonging to vulnerable groups such as “persons who have been subjected to torture”, must be considered to have special needs and to require specific support [3]. EU Member States are obliged to assess whether asylum applicants are indeed such vulnerable persons within a reasonable timespan after an application for international protection is made. EU directives are binding for EU Member States, requiring all EU Member States to establish the necessary structures for such an assessment. Suggestions for a comprehensive and routinely performed assessment have been made [4], but most often, if at all, countries tackle this challenge via temporary projects [5].

The in:Fo-project (short for German “interdisziplinär: Folterfolgen erkennen und versorgen”) was launched to counteract a lack of structures in Germany and to optimize the medical and psychosocial support of persons with a history of torture, including the assessment of the experienced violence according to the Istanbul Protocol (IP) [6]. The project was funded by the AMIF, the European Asylum, Migration and Integration Fund, and extended from July 1, 2018, to June 30, 2020. By building up a dedicated network of professionals to enable such assessments on a regional level, it was meant to provide insights and serve as a best practice model.

In:Fo included a multi-professional approach that aimed on improving

- the identification of persons with torture experience by training medical staff in shelters for asylum seekers,
- the clarification of their individual needs by establishing a case management system,
- the assessment of the alleged torture experience by following the guidelines of the IP and
- the access to medical and psychosocial support-institutions.

The IP is the international guideline for the investigation and documentation of torture. It advocates an interdisciplinary approach comprising a (forensic) medical examination as well as a psychological appraisal.

As the forensic evaluation of traumatological findings is a key duty of Institutes of Legal Medicine in Germany, it is only reasonable to include such expertise in these assessments. However, although the IP has already been published in 1999, it is still rather unknown [7–11]. In Germany, forensic medical expert reports in the context of claimed torture have only rarely been written in the past. Even more, the aspect of interdisciplinarity and collaboration with psychiatric/psychological experts has been neglected.

In this respect, the in:Fo-project represented an absolute novelty with regard to the assessment of alleged torture. The forensic physical evaluation was covered by expert physicians at the Institute of Legal Medicine in Düsseldorf, whereas the psychological appraisals were organized by three participating psychosocial/psychiatric facilities: the Psychosocial Centre for Refugees in Düsseldorf, the Medical Refugee Help Centre in Bochum and the Transcultural Day Care Unit at the Düsseldorf Clinic for Psychiatry and Psychosomatic Medicine (for easier reading, all three will be referred to as PSCs from here on).

As part of the case management process, each study participant was first assessed at one of the participating PSCs. Based on the information gathered during an initial interview and—depending on availability and need—a consultation with a physician at said PSC for a general medical appraisal, a decision was made whether a forensic medical examination was likely to further the clarification of facts. Study participants presenting for a forensic medical examination mostly received a comprehensive forensic medical expert opinion; only in single cases merely a forensic medical report of findings was prepared. Language and culture mediators were provided whenever necessary.

While there have been comparable programs in other countries [12–15], the in:Fo-project is Germany’s first large-scale attempt to compile expert opinions following the standards set by the IP. The focus of this publication lies primarily on the forensic medical aspects, especially their significance in the context of an interdisciplinary clarification of facts as proposed by the IP guidelines. We set out to examine whether this unprecedented degree of “forensic medical input” had measurable effects on asylum proceedings. In order to do so, we reviewed the project cases with regard to a possible “connection” between certain characteristics and the progress of the individual asylum proceeding.

## Methods

The methodological approach was based on a master’s thesis of one of the co-authors, submitted to the Department of Psychology at the University of Cologne [16]. However, the presented results are completely original since new statistical calculations, based on a larger data set, had been performed. The study was approved by the local ethics committee (study number 2022–1869).

**Fig. 1:**
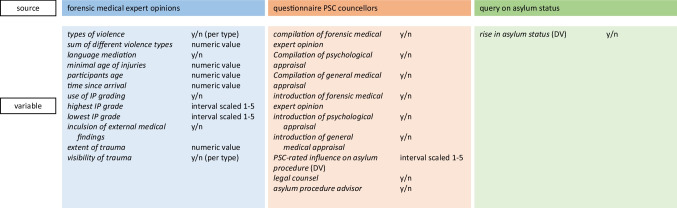
Overview of variables used for statistical analysis with their encoding and the respective data sources. Dependent variables = DV. Dichotomous variable encoded yes or no = y/n. Continuous variables = numeric value. Interval scaled variables with values 1 to 5 = interval scaled 1–5. Psychosocial Centre for Refugees = PSC. Istanbul Protocol = IP

Different variables that might have influenced the study participants’ asylum procedures were drawn from (a) the forensic medical documentation, (b) a questionnaire for PSC counsellors and (c) a query on the asylum status of the study participants. In the following, all variables used are explained in detail and displayed in *italic* characters. An overview is presented in Fig. 1.

### Study participants

**Fig. 2:**
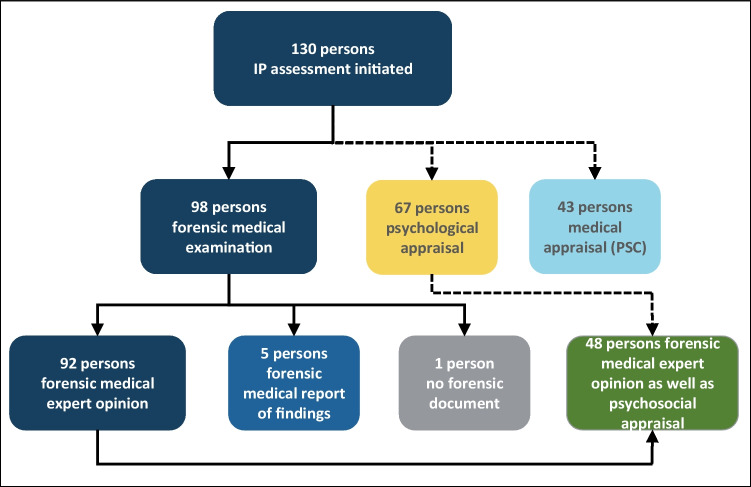
Overview of study participants. Istanbul Protocol = IP. Psychosocial Centre for Refugees = PSC

The participants of the in:Fo-project that are included in the study can be categorized as shown in Fig. 2.

When entering the project, they underwent an extensive assessment of their individual needs. Forensic medical examinations were offered whenever deemed necessary to clarify stated torture experiences. Thus, expert reports were never commissioned by official authorities but were treated as private commissions and handed over to the study participants’ legal counsellors.

Most of the examinations resulted in a full forensic medical expert opinion. Whenever feasible, they included an IP grading at the discretion of the forensic medical experts involved.

In six cases, a forensic medical expert opinion was deemed irrelevant to the proceedings by the study participants’ legal counsellor after the examination had already been performed. Therefore, the forensic medical documentation was reduced to a simple report of findings (including only the stated history and the examination findings). In one case, even such a shortened documentation was rejected.

In 43 cases, a medical appraisal was performed by physicians in the PSCs. These examinations aimed at a rather general physical and psychosocial assessment and were not equivalent to the more specialized forensic medical expert opinions and the psychological appraisals.

### Evaluation of forensic medical expert opinions/reports of findings

All forensic medical expert opinions and reports of findings were retrospectively scrutinized, regarding:

- *Types of violence* (see Table 1 for definitions) specified in each case
- *Sum of different violence types*
- *Use of IP grading*
  - Was the IP grading system applied?
  - If yes: which was the highest (*highest IP grade*) and which was the lowest (*lowest IP grade)* grade given?
- *Inclusion of external medical findings*
  - Was there explicit mention of other medical findings (e.g. radiological, dental, orthopaedic)?
- *Extent of trauma*
  - Number of separate entries regarding injuries
  - Number of pages

**Table 1:** Definitions and rates of mention of different types of violence during forensic medical examinations

Type of violence	Definition	*n*	Percentage
Blunt force trauma: object	Use of objects to inflict blunt force—excluding both whip-like objects and deliberate strikes against the foot soles	87	88.80%
Blunt force trauma: hand	Use of hands to inflict blunt force	58	59.20%
Blunt force trauma: kick	Use of feet to inflict blunt force	47	48.00%
Thermal	Use of temperature to inflict injuries and/or pain	38	38.80%
Sharp force trauma	Use of objects to inflict sharp force	35	35.70%
Positional	Enforcement of prolonged and/or distorted body positions to inflict injuries and/or pain. [May overlap with severe binding.]	32	32.70%
Sexualized	Forced and/or violent sexual act	29	29.60%
Severe binding	Use of restraints that is prolonged and/or inflicts injuries. [May overlap with positional violence.]	28	28.60%
Electrical	Use of electric currents to inflict injuries and/or pain	21	21.40%
Blunt force trauma: falanga	Use of objects to inflict strikes against the foot soles	17	17.40%
Blunt force trauma: whipping	Use of flexible, lengthy objects to inflict blunt force—excluding deliberate strikes against the foot soles	16	16.30%
Other	Other actions capable of inflicting physical injuries and/or pain, such as chemical torture, noise torture	14	14.30%
Shot	Use of firearms to inflict injuries	12	12.20%
Asphyxia	Deliberate decrease of oxygen supply	8	8.20%
Extraction	Forceful extraction of body parts (teeth, nails, etc.)	8	8.20%
Female genital mutilation (FGM)	Deliberate injuries to the vulva without medical indication	5	5.10%
Explosion	Use of explosive devices to inflict injuries	3	3.10%

Certain specifics concerning the examination situation, the anamnesis and the study participant were also covered:

- *Language mediation*
  - Language and culture mediator present during the forensic examination?
- *Minimal age of injuries*
  - Number of months between the most recent injury (as reported by the participants) and the forensic medical examination
- *Participant’s age*
  - Age at the time of the forensic examination
- *Time since arrival*
  - Number of months between arrival in the European Union and the forensic examination

The forensic medical expert opinions were also reviewed concerning easily recognizable injuries that might possibly influence the decision-making process on the part of German authorities:

- *Visibility of trauma*
  - Visible trauma residues in the facial area?
  - Visible disfigurements, amputations, etc.?
  - Obvious and easily noticeable loss of a body function (e.g. pronounced walking impairment)?

### Questionnaires for PSC counsellors

Professional opinions of the responsible PSC counsellors were gathered to operationalize the proceedings for statistical analysis.

Following the end of the project duration, questionnaires were sent out for all 130 cases. PSC counsellors had to rate the impact of the participants’ inclusion into the in:Fo-project with its assessment process.

Counsellors were first asked to indicate both how distressful and how helpful the inclusion had been—from their client’s point of view. Secondly, they were asked to do so from their own professional perspective, with further emphasis on whether inclusion had been helpful regarding diagnostic and therapeutic aspects. They were given the option to elaborate on this via free-text answers.

They also stated whether any expert opinion had been prepared as part of the assessment process (*compilation of forensic medical expert opinion, … of psychological appraisal, … of general medical appraisal*) and whether any of them had actually been introduced into the asylum procedure (*introduction of forensic medical expert opinion, … of psychological appraisal, … of general medical appraisal*).

Counsellors were then asked to judge from their subjective point of view whether these expert opinions influenced the asylum procedure upon their introduction: besides a positive or negative influence on the protection status, this also included, for example, the assignment of an appropriate decider [17], specifically trained for cases of alleged torture. The answers represent the dependent variable (DV) *PSC-rated influence on asylum procedure*.

Finally, they were asked to specify whether a *legal counsel* and/or an *asylum procedure advisor* had been involved in each case.

### Query on asylum status

As a follow-up, independent of the above-mentioned questionnaire, the responsible PSC counsellors were also asked to review IP assessment cases to determine, if there had been an objective gain (i.e. higher status) or not (i.e. identical or lower status) regarding the asylum status since entry of the participant into the project. This constituted the dichotomous DV *rise in asylum status*.

Asylum status was differentiated as follows from “highest” to “lowest”:

- Settlement permit
- Refugee protection
- Subsidiary protection
- Deportation ban
- Residence authorisation
- Short-term permit (permission to remain until deported) or undocumented

### Statistical analyses

All calculations were performed using the “jamovi” software (www.jamovi.org), including logistic/linear regression and bivariate analysis. Two different operationalizations were chosen:

#### Analysis of DV *PSC-rated influence on asylum procedure*

We tested if the introduction of expert opinions had a measurable influence on the asylum proceedings. Via linear regression, we calculated whether their introduction predicted a perceived influence on the proceedings from the responsible PSC counsellors’ point of view. Three dummy-coded independent variables (*introduction of forensic medical expert opinion/psychological appraisal/general medical appraisal*) were modelled as predictors. We separately controlled for these independent variables. More complex calculations regarding a possible influence of the introduction of more than one report/appraisal were not feasible due to small numbers.

#### Analysis of DV *rise in asylum status*

We calculated via logistic regression whether one or more independent variables could be used to predict a heightened asylum status. The independent variables were used as listed in Fig. 1, the only addition being that *introduction of a forensic medical expert opinion* was controlled for with *highest IP grade* as a covariant.

#### Bivariate analysis

We also performed discovery-driven bivariate analysis regarding the abovementioned DVs.

For the interval-scaled DV *PSC-rated influence on asylum procedure*, this included a correlation matrix with the following variables:

- *Highest IP grade*
- *Lowest IP grade*
- *Minimal age of presented injuries*
- *Participant’s age*
- *Extent of trauma*
- *Total sum of different violence types*

For the dichotomous DV *rise in asylum status*, this included chi-square tests with the following variables (which could not be included in the abovementioned regression analysis due to small numbers):

- *Legal counsel*
- *Asylum procedure advisor*
- *Stage of the asylum procedure*

## Results

### Demographic and chronological information as derived from the forensic documents

Eighty-seven participants identified themselves as male and 11 participants as female. Ages ranged from 16 to 63 years (mean age: 31.0 years). Countries of origin varied between 27 countries; most frequent were Guinea (19.4%), Sri Lanka (12.2%) and Iraq (7.1%). Language mediation was necessary in 82.7% of the cases, most often for Arabic (11.2%), Tamil (11.2%) and Farsi/Dari (10.2%).

Based upon the stated history, the minimal age of the presented scars ranged between 10 and 240 months (mean ≈ 65 months). The participants had arrived in the European Union between 6 and 141 months (mean ≈ 38.5 months) prior to the examination.

Most of the participants had a residence authorisation when entering the project.

### Types of violence, medical information and IP grading as derived from forensic medical expert opinions

Blunt force trauma was by far the most frequently named form of violence and was mentioned in a total of 96 cases (≙ 98,0%), with objects (88.8%) and hands/fists (59.2%) as most frequent vectors (Table 1). Other common types of violence included thermal violence (38.8%) and sharp force trauma (35.7%).

The sum of different types of violence varied markedly between cases. While 17.3% of individuals reported only one type of violence, other cases included up to eight different types of violence (Table 2).

**Table 2:** Sums of different types of violence mentioned during forensic medical examinations

Number of types of violence	*n*	Percentage
1	17	17.30%
2	23	23.50%
3	13	13.30%
4	20	20.40%
5	11	11.20%
6	8	8.20%
7	4	4.10%
8	2	2.00%

As for the trauma extent, the length of forensic expert opinions varied between 4 and 14 pages (mean ≈ 7.91), while the number of separate entries varied between 1 and 76 (mean ≈ 22.12).

Visible trauma residues in the facial area could be discerned in 50 cases, visible deformities in 12; an obvious loss of function was present in 17 cases.

External medical documentation was included in 33 of the forensic medical expert opinions.

IP grading was applied in 60 of the 92 forensic medical expert opinions. Grades were given as follows:

- 39 cases were graded as “consistent with”
- 12 cases were graded as “highly consistent”
- 4 cases were graded with varying IP grades and “highly consistent” as highest grade
- 5 cases were graded with varying IP grades and “typical of” as highest grade

The remaining 32 forensic medical expert opinions abstained from an IP grading and included an individually worded plausibility check instead. The proportion of expert opinions without IP grade increased notably over the course of the project: while the first 6-month-period saw only 7.1% of expert opinions without IP grading, the fourth and final 6-month-period included 58.3% of such expert opinions.

### Questionnaire results

Final questionnaires were filled out by the responsible PSC counsellors at least partly in 119 cases (≙ 91.5% of all IP assessment cases), out of which 81 cases had also received a forensic medical expert opinion. Table 3 shows how the counsellors rated the impact of inclusion into the in:Fo-project and its subsequent assessment process on the study participants. The process was mostly deemed distressful but also diagnostically/therapeutically helpful. Counsellors elaborated on this via free-text-items with statements such as “being seen”, “being taken seriously”, “normalisation of the experience” and “acknowledgement of suffering” from their clients’ perspective, as well as “ways out of speechlessness”, “detabooisation” and “insight” regarding therapeutic helpfulness.

**Table 3:** Evaluation of the final questionnaires for Psychosocial Centre for Refugees (PSC) counsellors presenting the results for a possible impact of the inclusion in the in:Fo-project and the assessment process on study participants (A to E) and the PSC-rated influence on asylum procedure of the expert opinions (F)

Questionnaire item	Response	*n*	Percentage
A)			
Distressful to the client from clients’ point of view	No	6	5.30%
*n* = 114	Rather no	25	21.90%
	Rather yes	42	36.80%
	Yes	41	36.00%
B)			
Distressful to the client from counsellors’ point of view	No	10	8.80%
*n* = 113	Rather no	18	15.90%
	Rather yes	37	32.70%
	Yes	48	42.50%
C)			
Helpful to the client from clients’ point of view	No	1	0.90%
*n* = 109	Rather no	17	15.60%
	Rather yes	59	54.10%
	Yes	32	29.40%
D)			
Diagnostically helpful to the client from counsellors’ point of view	No	2	2.20%
*n* = 92	Rather no	18	19.60%
	Rather yes	46	50%
	Yes	26	28.30%
E)			
Therapeutically helpful to the client from counsellors’ point of view	No	0	0%
*n* = 59	Rather no	6	10.20%
	Rather yes	39	66.10%
	Yes	14	23.70%
F)			
*PSC-rated influence on asylum procedure*	Not at all	19	16.00%
*n* = 119	Rather no	6	5.00%
	Unclear	48	40.30%
	Rather yes	10	8.40%
	Definitely	21	17.60%
	No answer (e.g. still pending, no contact with client)	15	12.60%

Table 3 also shows how the responsible PSC counsellors rated the DV *PSC-rated influence on asylum procedure*. Results were somewhat balanced, with a marked majority stating they were unclear on this topic.

### Results of queries on asylum status

A total of 62 project cases had been classified by the responsible PSC counsellors regarding a possible *rise in asylum status*. In 34 cases, the asylum status had undergone an improvement, in the remaining 28 cases, there had been no change or a downgrade in the asylum status.

In 50 cases, a classification was not possible because the asylum procedures were not yet completed.

### Results of statistical analysis

#### Results regarding DV *PSC-rated influence on asylum procedure*

The DV *PSC-rated influence on asylum procedure* was predicted by the *introduction of a forensic medical expert opinion* into the asylum procedure when controlling for *highest IP grade* (*p* = 0.016) (Table 4).

**Table 4:** Results of linear regression regarding the dependent variable *PSC-rated influence on asylum procedure* being predicted by the independent variable introduction of forensic medical expert opinion when (A) controlling for independent variable highest IP grade (*R*^2^ = 0.128) and (B) controlling for independent variable use of IP grading (*R*^2^ = 0,0759)

Predictor	Estimate	Standard error	*t*	*P*
A)				
Intercept	− 0.0972	0.847	− 0.115	0.909
*Introduction of forensic medical expert opinion*	18,353	0.737	2491	0.016
*Highest IP grade*	0.2583	0.276	0.937	0.353
B)				
Intercept	0.8554	0.531	1612	0.112
*Introduction of forensic medical expert opinion*	12,663	0.533	2377	0.020
*Use of IP grading*	− 0.0442	0.316	− 0.140	0.889

Moreover, the *introduction of forensic medical expert opinion* into the asylum procedure was considered influential when including the second predictor *use of IP grading* (*p* = 0.020) (also Table 4). Notably, the beta weight of the second predictor was negative.

The other calculations (linear regressions, bivariate analyses) did not yield any significant results regarding this DV.

#### Results regarding DV *rise in asylum status*

All analysis—both logistic regressions as well as chi-square tests—referring to the DV *rise in asylum status* did not yield statistically significant relations. Table 5 presents examples of the logistic regression calculations.

**Table 5:** Exemplary results of logistic regressions regarding dependent variable *rise in asylum status* with (A) predictor highest IP grade, (B) predictor introduction of a forensic medical expert opinion and controlling for highest IP grade and (C) predictor use of IP grading

Predictor	Estimate	Standard error	*Z*	*P*
A)				
Intercept	− 0.631	0.909	− 0.695	0.487
*Highest IP grade*	0.380	0.560	0.677	0.498
B)				
Intercept	− 17,516	2,399,545	− 0.007	0.994
*Introduction of a forensic medical expert opinion*	16,824	2,399,545	0.007	0.994
*highest IP grade*	0.475	0.577	0.823	0.411
C)				
Intercept	0.560	0.627	0.893	0.372
*Use of IP grading*	− 0.629	0.729	− 0.863	0.388

## Discussion

Despite inclusion of numerous items and extensive statistical analysis, our study did not reveal any significant correlations between the asylum status (DV *rise in asylum status*) and any of the other variables we examined; neither the given IP grade nor any other factor could be linked to a (positive) change in the asylum status.

However, at least from the subjective perspective of the PSC professionals involved, our results suggest that the introduction of a forensic medical expert opinion into an asylum procedure had an impact when a higher IP grade was applied. The opposite was the case when including the predictor *use of IP grading*. These results imply that forensic medical expert opinions were more likely to be perceived as having a favourable influence on the asylum procedure if they contained a higher IP grade or no IP grade at all, i.e. presenting only an individually worded plausibility check.

This finding is somewhat contradictory to other similar studies. In Italy, Franceschetti et al. [14] found a correlation between a favourable outcome of the asylum procedure and higher IP grades and the number of individual lesions/scars as well as certain types of violence (gunshot, sharp force). Aarts et al. [13] reported results from a Dutch study, demonstrating a significant correlation between the presence of physical symptoms and their consistency with the given story and the refugee status decision.

The differences to our study might have various reasons. We categorized some forms of violence differently compared to Franceschetti et al. [14]; also the extend of injuries was quantified based on the number of entries in the expert opinion. And while Aarts et al. [13] conducted statistical analysis based upon the final judicial outcome, we used the last known status of proceedings in comparison to the status at project entry. This was at least in part necessary because asylum procedures in Germany are often very lengthy, making a full follow-up very difficult and causing gaps in the data collection. In some cases, contact between the PSC counsellor and the study participant had been lost. Also, since we were not able to evaluate court files or any official documents, we have no knowledge about other factors apart from our reports/appraisals that might have been decisive for the final verdict.

**Fig. 3:**
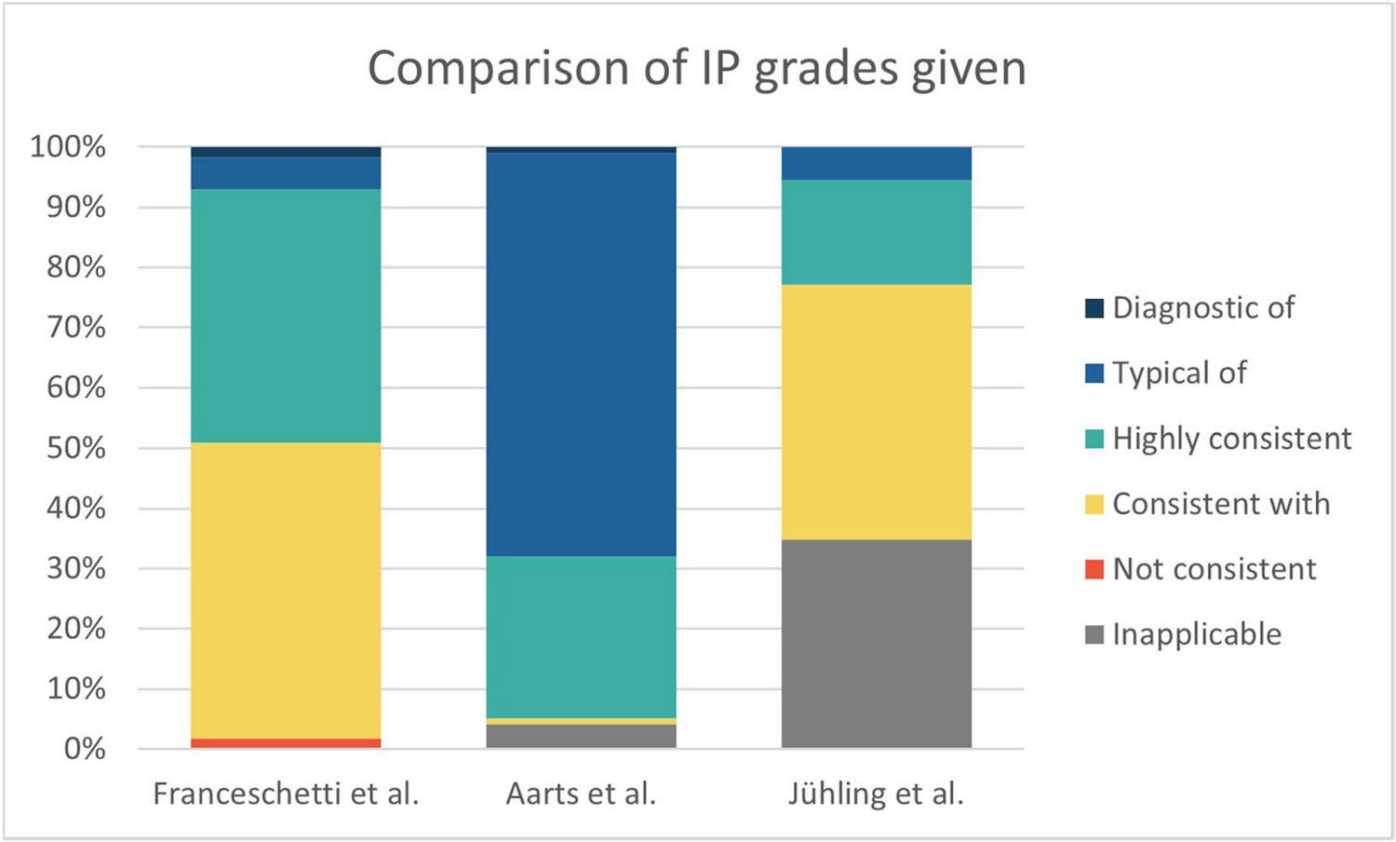
Comparison of IP grading between Franceschetti et al. [14], Aarts et al. [13] and the study at hand

Comparing the Italian, Dutch and our collective, we also found relevant differences regarding the rate at which certain IP grades were applied (Fig. 2). Like Franceschetti et al. [14], we applied the “consistent” grade most often (49.1% and 41.2%). Strong deviations can be seen when it comes to the IP grades representing higher levels of consistency—even more so when comparing to Aarts et al. [13] (Fig. 3).

This might be in part attributable to differences in the mean injury age. Due to its geographic position, Italy is a major point of entry to EU territory. Persons applying for asylum in Italy might present relatively shorter time spans between injury and (forensic medical) examination—which could result in a better detectability of at least some wounds. As far as the scar age could be retraced, none of the residues presented to us were younger than 10 months. This may have impacted the applicability of the IP grades as the ongoing healing process reduces the informative value of a wound [12]. Furthermore, the participants entered into the in:Fo-project typically in an advanced state of their asylum procedure. Very “obvious” cases—which would have received a high IP grade—were maybe already “filtered out” and had attained full asylum status or the like before even contacting a PSC, thereby bypassing the in:Fo-project completely. Also, some persons that would have been eligible for a forensic medical examination declined the offer. Conceivably this concerned severe/obvious cases at a disproportionate rate.

It is quite possible that such external factors could have attributed to a selection bias in our sample population. Even so, the findings in our study raise the question whether international standards such as the IP can be used uniformly under different conditions and whether the assigned IP grades are internationally comparable.

Nonetheless, since a general positive effect of the introduction of forensic medical expert reports in asylum procedures can be derived from our results, this study supports the request of a widened application of the IP standards in cases of alleged torture. Besides objective impacts, also subjective gains on a diagnostic and therapeutic level have been stated by the PSC counsellors—although the evaluation process was qualified as being stressful for the study participants.

However, (forensic) medical experts must be careful with the simplified, schematic evaluation that is proposed by the IP grades. An informal query among forensic physicians involved in the project identified a “feeling” that the IP grading system did not always adequately reflect the complexity of the cases. The presented torture sequelae had in virtually all cases already undergone full cicatrisation, which hampered an in-depth assessment of injury characteristics and most often precluded high IP grades.

It seems that the IP grading system reaches its limits when dealing with persons that suffered from torture a long time ago, though it might certainly help to standardize and simplify the evaluation of injuries shortly after the torture event (circumstances, for which it was primarily developed) and especially for clinicians with a lack of forensic experience and training. In cases in which only scars are left for evaluation, our findings suggest that an individually worded assessment, ideally done by a forensically experienced physician, should be preferred. The importance of a particular training has been shown before [18], while other authors also reported a modification of the IP grading system [19]. Also, the relevance of an additional psychological appraisal must be underlined. Although an impact on asylum procedures could not be detected statistically in our study, especially in cases in which physical wounds have completely healed and “disappeared” or never existed from the start, an evaluation of psychological consequences of torture is of even greater importance [20].

## Limitations

This publication is subject to some limitations. The data used was gathered from various sources which may have led to discrepancies regarding definitions, categorizations and the number of items. Some cases were primarily handled by external PSCs, who coordinated the entry into the project and the follow-up communication. Those cases often suffered from a reduced reply rate. In some cases, contact to the study participant was lost. Some persons who would have been eligible for referral to the Institute of Legal Medicine declined the offer. A final asylum status could not be determined in all cases since asylum proceedings were not always completed. Several potentially relevant factors were not easy to operationalize (e.g. country of origin: a potential variable would have resulted in 27 values, precluding statistical calculations due to small sample sizes). Lastly, one of the operationalized DVs was dichotomous, which restricted the available statistical options, possibly concealing some underlying associations.

## Conclusion

The evaluation of the first large-scale attempt in Germany to implement the IP recommendations came up with some unexpected results. Effects on asylum proceedings and the consequent asylum status could be found when a forensic medical expert opinion was introduced and if (a) persons presented considerable injuries resulting in a high IP grading or if (b) the forensic expert opinion abstained from an actual IP grade in favour of an individually worded approach. This raises questions regarding the use of the IP grading system in differing scenarios. Though this easy-to-handle approach is recommendable especially for forensically unexperienced physicians in cases of recent torture events, it reaches its limits when examining persons that suffered from torture a long time ago. Under such difficult circumstances, the evaluation should be performed by an experienced forensic physician who should rely on his/her own words. Apart from that, the expert reports should follow the recommendations of the IP and further efforts must be made to make the IP known, since favourable effects were not only detected with regard to the asylum proceedings, but also with a view to the psychosocial well-being of the study participants.

## Data Availability

The data that support the findings of this study are available from the corresponding author, F. M., upon request.
